# Supplementary material for: Self-trapped state enabled filterless narrowband photodetections in 2D layered perovskite single crystals
Source: Nat Commun. 2019 Feb 18;10:806. doi: 10.1038/s41467-019-08768-z (PMC6379360; doi:10.1038/s41467-019-08768-z)
Supplement: Supplementary file 1 — Supplementary Information [file 41467_2019_8768_MOESM1_ESM.pdf]

Supplementary Information for:

Self-trapped state enabled filterless narrowband photodetections in 2D layered perovskite single crystals

Junze Li[1], Jun Wang[1], Jiaqi Ma[1], Hongzhi Shen[1], Lu Li[1], Xiangfeng Duan[2], and Dehui Li[1, 3*]

[1]*School of Optical and Electronic Information, Huazhong University of Science and Technology, Wuhan, 430074, China;*

[2]*Department of Chemistry and Biochemistry, University of California, Los Angeles, California 90095, USA;*

[3]*Wuhan National Laboratory for Optoelectronics, Huazhong University of Science and Technology, Wuhan, 430074, China;*

*Correspondence to: Email: dehuili@hust.edu.cn.

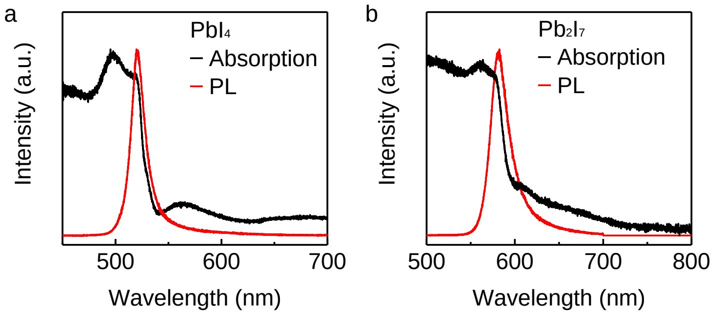

Supplementary Figure 1. Absorption and PL spectra of $(BA)_2PbI_4$ **(a)** and $(BA)_2(MA)Pb_2I_7$ **(b)** microplates with thickness about 100 nm at room temperature.

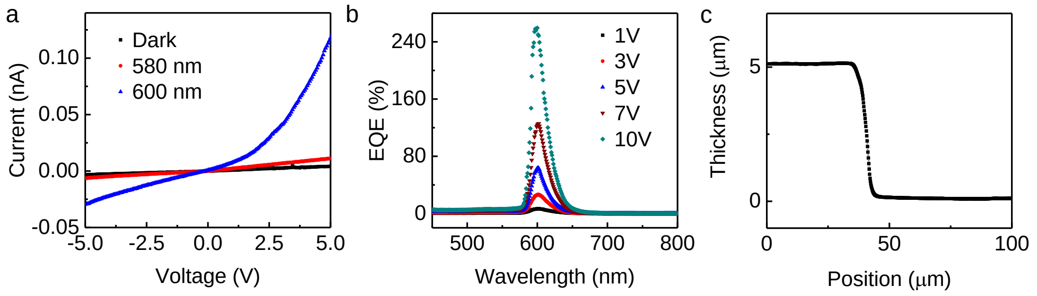

Supplementary Figure 2. (a) Dark current and photocurrent of a 5-μm-thick $(BA)_2(MA)PbI_4$ device with Config.1 under a 580 (600) nm monochromatic illumination with a power density of 12 (16) μW cm^{-2}. **(b)** EQE spectra of the 5-μm-thick $(BA)_2(MA)Pb_2I_7$ device under different biases. **(c)** Thickness profile of the 5-μm-thick $(BA)_2(MA)Pb_2I_7$ device.

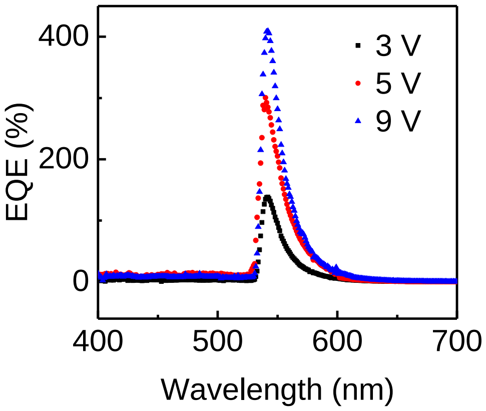

Supplementary Figure 3. EQE spectra of the a 100-μm-thick $(BA)_2PbI_4$ vertical device (Config.1) under different biases.

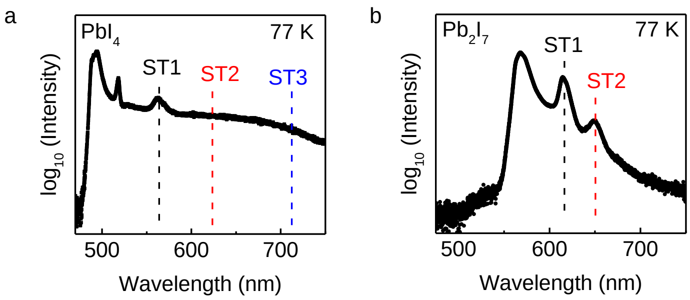

Supplementary Figure 4. Low temperature PL spectra of $(BA)_2PbI_4$ **(a)** and $(BA)_2(MA)Pb_2I_7$ **(b)**. Plotted in *log* scale to magnify weak self-trapped (ST) states peaks. ST1, ST2 and ST3 represent the different type of self-trapped states probably formed at different sites.

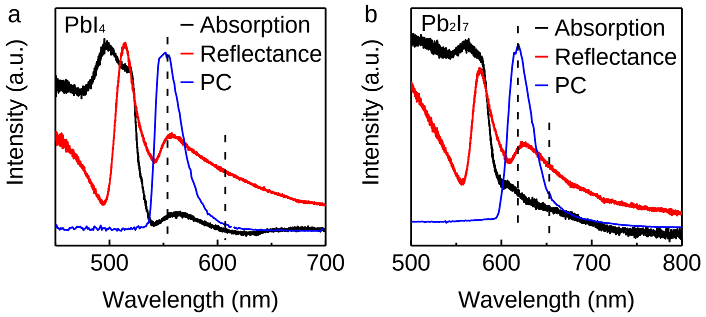

Supplementary Figure 5. Absorption, reflectance and photocurrent spectra of $(BA)_2PbI_4$ **(a)** and $(BA)_2(MA)Pb_2I_7$ **(b)**. The photocurrent peak marked by dash line matches with ST1 in absorption and reflectance spectra. We intentionally used exfoliated microplates with thicknesses about 100 nm so that the absorption would not saturate at the free exciton position and above the bandgap regime. Under such case, the weak self-trapped state induced absorption might be observed. In bulk crystals, the absorbance can rapidly change over several orders of magnitude near the free exciton absorption peak and thus the weak self-trapped state induced absorption is completely suppressed and invisible in the absorption spectrum.

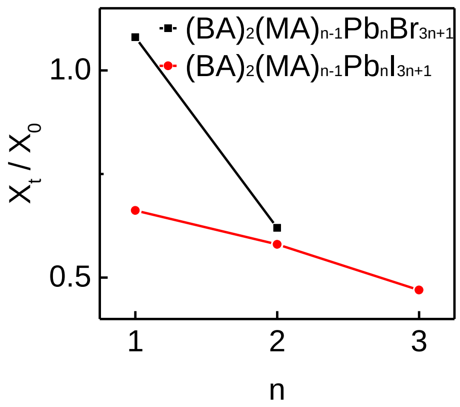

Supplementary Figure 6. Normalized intensity ratio of self-trapped state associated reflection peak to the free exciton associated reflection peak as a function of the layer number *n*.

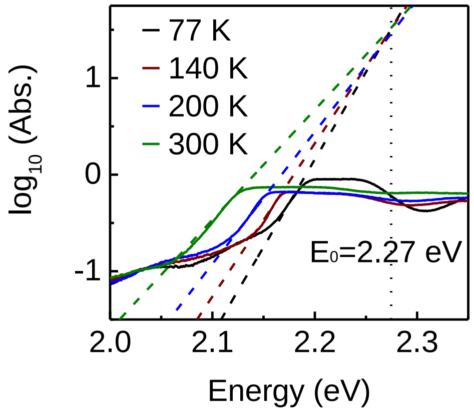

Supplementary Figure 7. Temperature dependent absorption of $(BA)_2(MA)Pb_2I_7$ and Urbach edge fitting of different temperature are plot in dash lines.

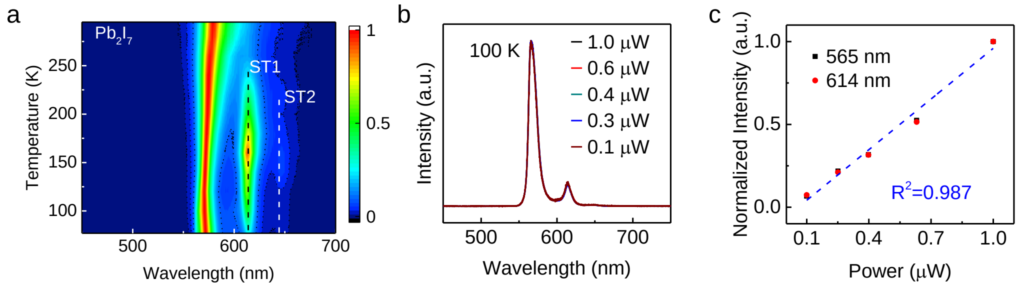

Supplementary Figure 8. (a) Temperature-dependent PL spectra of $(BA)_2(MA)Pb_2I_7$. **(b)** Normalized PL spectra under different incident laser power. **(c)** Normalized PL peak intensity of free exciton (565 nm) and ST1 exciton (614 nm) versus excitation power.

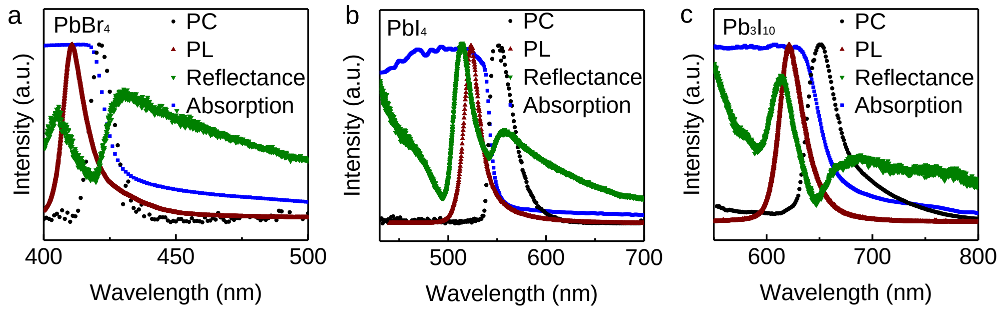

Supplementary Figure 9. Normalized PC, PL, reflectance and absorption spectra of the devices based on $(BA)_2PbBr_4$ **(a)**, $(BA)_2(MA)PbI_4$ **(b)** and $(BA)_2(MA)_2Pb_3I_{10}$ **(c)**, respectively. For $(BA)_2(MA)Pb_2Br_7$, $(BA)_2(MA)_2Pb_4I_{13}$ and $(BA)_2(MA)_2Pb_5I_{16}$, due to the presence of the impurity phases, we did not show the comparisons here.

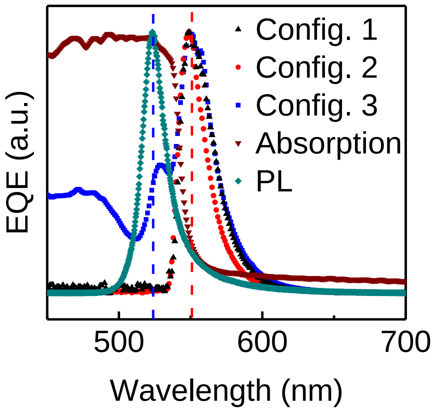

Supplementary Figure 10. Normalized EQE spectra with different measurement configurations, PL and absorption spectra of $(BA)_2PbI_4$.

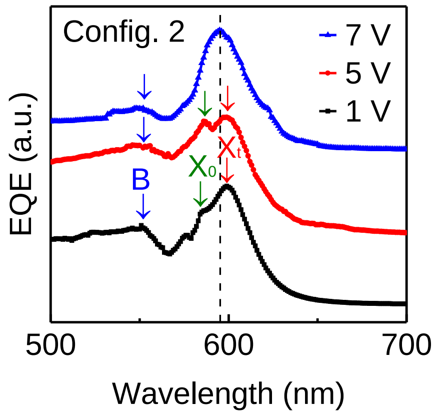

Supplementary Figure 11. Normalized EQE spectra of a 10-μm-thick device (Config. 2) under different biases. X_0 and X_t merge into one peak as the bias increases.

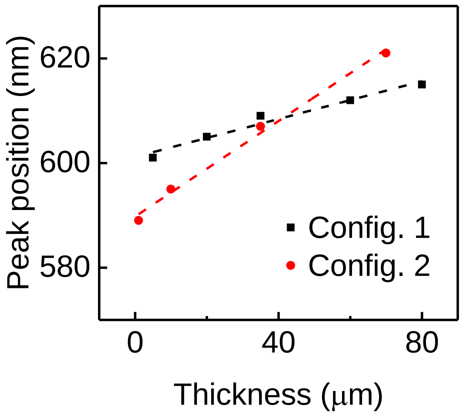

Supplementary Figure 12. Thickness dependence of peak position for $(BA)_2(MA)Pb_2I_7$ vertical and lateral devices under the same applied electrical bias extracted from the EQE spectra in Fig. 4g and 4h.

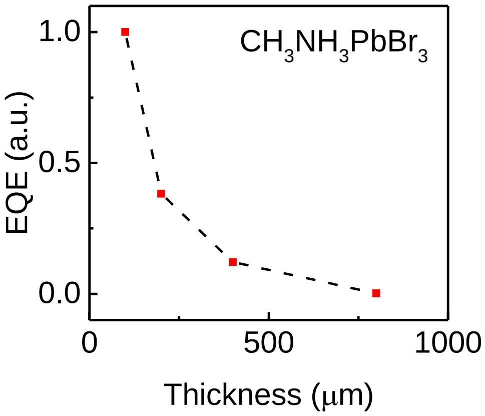

Supplementary Figure 13. Normalized EQE of 3D perovskite under the same applied electrical field. The data points are taken from previous work [10].

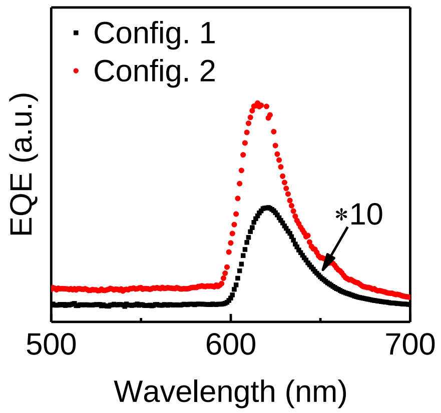

Supplementary Figure 14. EQE spectra of an 80-μm-thick $(BA)_2(MA)Pb_2I_7$ device with different configurations. Config. 2 shows a much larger EQE than that of Config. 1.

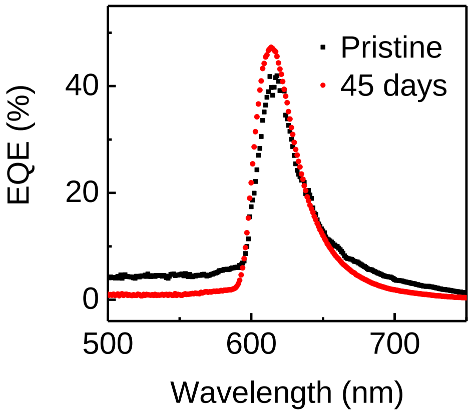

Supplementary Figure 15. EQE spectra of a pristine $(BA)_2(MA)Pb_2I_7$ vertical device (Config. 1) and the same device after being stored under ambient conditions for 45 days. Measurements were carried out under a bias of 3 V.

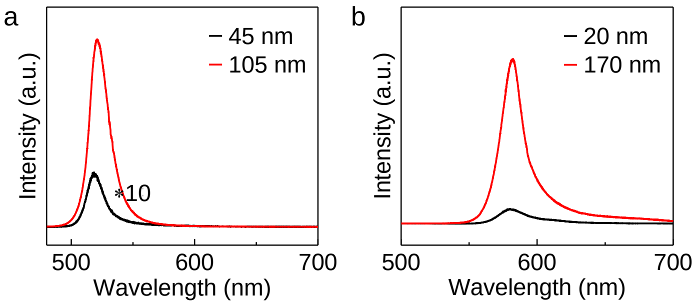

Supplementary Figure 16. PL spectra of the $(BA)_2PbI_4$ **(a)** and $(BA)_2(MA)Pb_2I_7$ **(b)** perovskite plates with different thicknesses at room temperature. Emission is significantly quenched in a thinner plate, suggesting the exciton ionization near the surface.

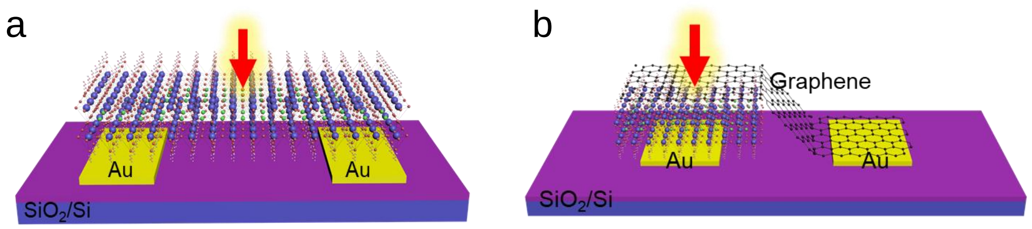

Supplementary Figure 17. Schematic illustrations of the thinner plate two-probe lateral (**a**) and vertical (**b**) devices.

Materials	Response Peak (nm)	FWHM (nm)	EQE (%)	Detectivity (Jones)	Electric Field (V μm^{-1})	Ref.
$MAPbBr_3$	570	35	20	-	0.01	10
$MAPbI_{3-x}Br_x$	Visible	< 100	12	$2*10^{11}$	1	11
$MAPbBr_{3-x}Cl_x$ $MAPbI_{3-x}Br_x$	Visible	< 20	1.6	$2*10^{10}$	0.008	12
$MAPbI_3$	830	33	10^4	10^{13}	0.025	13
$(BA)_2(MA)_{n-1}Pb_nBr_{3n+1}$ $(BA)_2(MA)_{n-1}Pb_nI_{3n+1}$	Visible	< 60	200	10^{11}	0.12	This Work

Supplementary Table 1. Performance comparison between representative filterless narrowband photodetectors based on perovskite via CCN strategy.

Supplementary Note 1: The presence of the self-trapped states in our 2D perovskites

The formation of the self-trapped states is due to the strong electron-phonon interaction, which would introduce the lattice distortion and thus trap carriers and/or excitons within the potential wells. In 2D perovskites, previous studies revealed that the self-trapping introduces the extra absorption and emission peak below bandgap and thus greatly alter the electrical and optical properties of the 2D perovskites including the thermal activated broad white light emission [1] and hopping transport of charge carriers [2]. Also, for some sorts of 2D perovskites, multiple broad emission peaks from self-trapped states have been observed originated from self-trapping of electrons, self-trapping of holes and/or self-trapping of both electrons and holes, which gives the different trapping energies and thus different emission peaks [3]. In $(BA)_2(MA)_{n-1}Pb_nI_{3n+1}$ series, the self-trapped states have been also observed in previous report, supported by the sub-bandgap emission peak at low temperatures and room-temperature transient and steady-state absorption spectra [4-6]. We have carried out a series of experiments to verify that the self-trapped states are formed in our 2D perovskite series.

First, we have carried out the temperature dependent micro-photoluminescence (μ-PL) and micro-absorption measurements on the exfoliated microplates. We intentionally used exfoliated microplates with thicknesses of about 100 nm so that the absorption would not saturate at the free exciton band and above the bandgap regime. Under such case, the weak self-trapped state induced absorption might be observed. In bulk crystals, the absorbance can rapidly change over several orders of magnitude near the free exciton absorption peak and thus the weak self-trapped state induced absorption is completely suppressed and invisible in the absorption spectrum, as shown in Fig. 1b in the maintext.

Previous study has reported that the PL spectra of the $(BA)_2PbI_4$ exhibit three distinguish peaks below the free exciton at 4K [5]. The power dependent spectra show that all those three peaks exhibit a nearly linear dependence on the excitation power, indicating an intrinsic origin of these emission peaks rather than defects or bi-excitons [5]. Thus, both peaks might be due to the self-trapped states but formed at different sites (*e.g.* Pb_2^{3+}, Pb^{3+} and I^{2-}) according to previous report [3]. Similar spectrum also has been reported in organic material, *e.g.* perylene, from which rather narrow emission peaks due to the transition from the lowest self-trapped states to the intramolecular vibrational levels [7].

Supplementary Figure 4a and 4b display the PL spectrum for $(BA)_2PbI_4$ and $(BA)_2(MA)Pb_2I_7$, respectively. Based on above discussion, we attributed the emission peaks below free exciton more than 100 meV in $(BA)_2PbI_4$ microplates (Supplementary Fig. 4a) to the different type of self-trapped (ST) states, and labelled them as ST1, ST2 and ST3 according to the previous reports [4,5]. In $(BA)_2(MA)Pb_2I_7$ microplates (Supplementary Fig. 4b), we only observe two peaks below free exciton and the third one may be too weak to be observed at 77 K. In absorption spectrum, the ST1 associated absorption peak has also been observed while ST2 peak shows a rather weaker intensity than ST1 for both $(BA)_2PbI_4$ and $(BA)_2(MA)Pb_2I_7$ microplates (Supplementary Fig. 5). Similar absorption spectrum also has been reported in thin film by previous work and assigned to the self-trapped state induced absorption as shown in previous work [6]. Remarkably, the absorption peak of

ST1 can sustain up to room temperature and matches very well with the reflection peak of self-trapped states and the narrowband photoresponse peak (Supplementary Fig. 5a and 5b), suggesting that they originate from the same optical transition: self-trapped states. Furthermore, we also extracted the intensity ratio of self-trapped states associated reflection peak to the free exciton associated reflection peak and found that the ratio continuously decreases with the increase of the layer number n (Supplementary Fig. 6), which is another evidence for the presence of the self-trapped states since the trap states prefer to be localized at the interfaces of 2D perovskites [4].

Second, we have measured the temperature dependent absorption spectra of $(BA)_2(MA)Pb_2I_7$ sample and extracted the Urbach slope to confirm the existence of the self-trapped states in our samples. Due to the coexistence of the mixed phases at low temperature in $(BA)_2PbI_4$ sample, we only focus on the $(BA)_2(MA)Pb_2I_7$ samples. Supplementary Fig. 7 shows the temperature dependent absorption spectra in semi-logarithmic scale together with the Urbach edge fittings. The Urbach tail can be expressed as $\alpha(E) = \alpha_0 \exp\left(-\sigma\frac{E_0-E}{kT}\right)$, where $\boldsymbol{\alpha}$ is the absorption coefficient, k is Boltzmann constant, T is the temperature, σ is the empirical slope coefficient, E_0 and $\boldsymbol{\alpha}_0$ are fitting parameters. Base on the Urbach slope parameter σ, a key parameter g (defined by $\sigma = s/g$, where $\boldsymbol{s}$ is direct exciton edges determined from the numerical simulations (1.5 for 3D and 1.24 for 2D)) is introduced to characterized the electron-phonon coupling strength and thus used to predict the existence of self-trapped states. If g exceeds a typical value, *e. g.* 0.92 for 3D and 0.87 for 2D, self-trapped states are prone to exist. By fitting the slope of the absorption edge, we obtain that $\sigma = 0.6825$, $g = 1.817$ (lager than 0.87) and $E_0 = 2.27$ eV for $(BA)_2(MA)Pb_2I_7$ samples [**8**]. Then, the Urbach energy E_u (defined by $E_u = kT/\sigma$) is calculated to be around 38 meV, which is close to previous reported value of 31 meV in $(BA)_2PbI_4$ [6]. This result also confirms that self-trapped states are present in our 2D perovskites.

Third, we demonstrated that the observed additional reflection peak and PL tails are not originated from permanent defects and impurity phases at least for $(BA)_2PbI_4$ and $(BA)_2(MA)Pb_2I_7$. To exclude the additional peak originated from permanent defect, we have carried out temperature- (Supplementary Fig. 8a) and power-dependent PL studies of $(BA)_2(MA)Pb_2I_7$ exfoliated microplates at low temperature (Supplementary Fig. 8b). From Supplementary Fig. 8a, we observe two peaks below free exciton at low-temperature and ST1 shows a much stronger intensity than ST2. Both ST1 and ST2 gradually become broaden and weak with the increase of the temperature and finally disappear at room temperature due to the reduced radiative recombination rate. The PL intensity of the additional emission peak shows a linear increase with the excitation power and the PL peaks show no shift with the excitation power (Supplementary Fig. 8c), suggesting the additional peak are not from permanent defects. Otherwise, the power different PL intensity should show different slope [5]. In addition, for all samples, the reflection spectra show similar spectral profile,

indicates the additional peak would be unlikely from the permanent defects. There should be no impurity phases for $(BA)_2PbI_4$ and our XRD pattern, absorption spectrum and PL spectrum all indicates that our $(BA)_2(MA)Pb_2I_7$ samples are quite pure. For $(BA)_2(MA)Pb_2Br_7$ samples,

the impurity phase has been identified as $(BA)_2PbBr_4$ phase, which would not introduce the below-gap states. Nevertheless, for *n*=3, 4 and 5 samples, the presence of the impurity phases would possibly lead to the below-gap states. To sum up, the self-trapped states lead to the additional reflection peak below the gap and PL tails for *n*=1 and *n*=2 samples and impurity phases would contribute to the below-gap states for $n > 2$ samples as well. Nevertheless, we believe that the self-trapped states still play the dominant role for the narrowband response in our devices since the impurity phases have rather strong optical transition strength compared with the self-trapped states and majority of the excitons generated within the impurity phases would recombine under such weak applied electric field in view of the energy band alignment which favors the energy funneling [9]. In contrast, the self-trapped states with a much longer lifetime could contribute to the photocurrent. Nevertheless, the presence of impurity phase would broad the FWHMs of the narrowband response, as has been observed in our experiment.

Supplementary Note 2: The role of self-trapped states in the narrowband photoresponse.

In this section, we will show that the narrowband response can indeed attribute to self-trapped states induced by strong electron-phonon interaction in our samples. First, if there is no contribution from the self-trapped states, there would be only two response peaks in Config. 3 devices. Rather, we observed three peaks with one response peak far below the free exciton peak (Fig. 4c). Provided that this X_t peak originates from the band tail absorption like 3D perovskite case, we should only observe the broadened free exciton peak rather than the distinctive X_t peak far below free exciton peak X_o. Second, without the contribution from the self-trapped states, the EQE of the narrowband response would continuously decreases with the increase of the thickness of the samples as 3D perovskite case [10] (Supplementary Fig. 13). Nevertheless, we observed that the EQE first increases and then decrease with the thickness of the samples under the same electric field (Fig. 4i), which agrees well with the ST1 peak in the reflection spectrum and absorption spectrum (Supplementary Fig. 5). This cannot be explained by bare exciton diffusion without considering the self-trapped states. Third, the narrowband response peak coincides well with the ST1 peak in absorption spectrum and reflection spectrum, suggesting that ST1 would contribute to the narrowband response. Furthermore, we expect that the EQE of 2D perovskite based devices would be much smaller than that of 3D counterparts if the regular band-tail states lead to the narrowband photoresponse, since the large resistance in the out-of-plane direction makes it more difficult for carriers to be extracted. In contrast, we observed an enhancement of EQE in 2D perovskite based narrowband photodetectors, which should result from the enhanced absorption due to the presence of self-trapped states. Based on the above discussion, we believe that the self-trapped states play an indispensable role in the narrowband photodetections in our 2D perovskite devices.

Supplementary Reference

1. Yangui, A. et al. Optical investigation of broadband white-light emission in self-assembled organic–inorganic perovskite $(C_6H_{11}NH_3)_2PbBr_4$. *J. Phys. Chem. C.* **119**, 23638-23647 (2015).

2. Straus, D. B. & Kagan, C. R. Electrons, excitons, and phonons in two-dimensional hybrid perovskites: connecting structural, optical, and electronic properties. *J. Phys. Chem. Lett.* **9**, 1434-1447 (2018).

3. Cortecchia, D. et al. Polaron self-localization in white-light emitting hybrid perovskites. *J. Matr. Chem. C.* **5**, 2771-2780 (2017).

4. Wu, X. et al. Trap states in lead iodide perovskites. *J. Am. Chem. Soc.* **137**, 2089-2096 (2015).

5. Blancon, J. C. et al. Scaling law for excitons in 2D perovskite quantum wells. *Nat. Commun.* **9**, 2254 (2018).

6. Ni, L. et al. Real-time observation of exciton-phonon coupling dynamics in self-assembled hybrid perovskite quantum wells. *ACS Nano* **11**, 10834-10843 (2017).

7. Nishimura, H., Yamaoka, T., Mizuno, K.-i., Iemura, M. & Matsui, A. Luminescence of free and self-trapped excitons in α-and β-perylene crystals. *J. Phys. Soc. Japan* **53**, 3999-4008 (1984).

8. Williams, R. T. & Song, K. S. The self-trapped exciton. *J. Phys. Chem. Solids* **51**, 679-716 (1990).

9. Cao, D. H., Stoumpos, C. C., Farha, O. K., Hupp, J. T. & Kanatzidis, M. G. 2D homologous perovskites as light-absorbing materials for solar cell applications. *J. Am. Chem. Soc.* **137**, 7843-7850 (2015).

10. Rao, H. S., Li, W. G., Chen, B. X., Kuang, D. B. & Su, C. Y. In situ growth of 120 cm^2 $CH_3NH_3PbBr_3$ perovskite crystal film on FTO glass for narrowband-photodetectors. *Adv. Mater.* **29**, 1602639 (2017).

11. Lin, Q., Armin, A., Burn, P. L. & Meredith, P. Filterless narrowband visible photodetectors. *Nat. Photon.* **9**, 687-694 (2015).

12. Fang, Y., Dong, Q., Shao, Y., Yuan, Y. & Huang, J. Highly narrowband perovskite single-crystal photodetectors enabled by surface-charge recombination. *Nat. Photon.* **9**, 679-686 (2015).

13. Saidaminov, M. I. et al. Perovskite photodetectors operating in both narrowband and broadband regimes. *Adv. Mater.* **28**, 8144-8149 (2016).
